# The Sequence of Intracranial Radiotherapy and Systemic Treatment With Tyrosine Kinase Inhibitors for Gene-Driven Non-Small Cell Lung Cancer Brain Metastases in the Targeted Treatment Era: A 10-Year Single-Center Experience

**DOI:** 10.3389/fonc.2021.732883

**Published:** 2021-10-14

**Authors:** Siran Yang, Jianping Xiao, Qingfeng Liu, Ye Zhang, Nan Bi, Xiaodong Huang, Xuesong Chen, Kai Wang, Yuchao Ma, Lei Deng, Wenqing Wang, Ruizhi Zhao, Junling Li, Junlin Yi, Shulian Wang, Yexiong Li

**Affiliations:** ^1^ Department of Radiation Oncology, National Cancer Center/National Clinical Research Center for Cancer/Cancer Hospital, Chinese Academy of Medical Sciences and Peking Union Medical College, Beijing, China; ^2^ Department of Medical Oncology, National Cancer Center/National Clinical Research Center for Cancer/Cancer Hospital, Chinese Academy of Medical Sciences and Peking Union Medical College, Beijing, China

**Keywords:** optimal sequence, hypofractionated stereotactic radiotherapy, targeted therapy, gene-driven, non-small cell lung cancer, brain metastases

## Abstract

**Purpose:**

The high intracranial efficacy of targeted therapeutic agents poses a challenge in determining the optimal sequence of local radiation therapy (RT) and systemic treatment with tyrosine kinase inhibitors (TKIs) in non-small cell lung cancer (NSCLC) patients with brain metastasis (BM). Therefore, we conducted a cohort study to elucidate the appropriate treatment strategy, either upfront RT or deferred RT including a toxicity assessment, in these patients.

**Patients and Methods:**

We retrospectively evaluated patients with gene-driven BMs from a single institution and divided them into deferred and upfront RT groups. Survival was estimated using a log-rank test. Intracranial progression was estimated using Fine-Gray competing risks model. Cox proportional hazards regression was performed for multivariable analysis in the entire group and subgroups.

**Results:**

Among the 198 eligible patients, 94 and 104 patients received deferred and upfront RT, respectively. The upfront RT group showed a lower intracranial progression risk with an adjusted sub-distribution hazard ratios of 0.41 (95% CI, 0.30–0.57) than did the deferred RT group (median intracranial progression-free survival [iPFS], 19.9 months *vs.* 11.1 months; *p* < 0.001). The median overall survival (OS; 43.2 months *vs.* 49.1 months, *p* = 0.377) and BM-specific survival (92.1 months *vs.* 82.9 months, *p* = 0.810) after salvage therapy were not significantly different between the upfront and deferred groups. Among patients with progressed extracranial disease, the deferred RT group showed significantly better OS than did the upfront RT group (44.0 *vs.* 28.1 months, *p* = 0.022). Grade 3–4 treatment-related adverse events were rare, and similar toxicities were observed between the two groups.

**Conclusion:**

Compared to the deferred RT group, the upfront RT group achieved longer iPFS and similar survival outcomes in most patients with gene-driven NSCLC BM, although patients with progression of extracranial disease might benefit from deferred RT. Both groups showed well-tolerated toxicities.

**Trial registration ID:**

NCT04832672.

## 1 Introduction

As systemic therapies have improved, brain metastases (BM) have been increasingly reported as one of the most common clinical events and causes of mortality in patients with advanced malignancies, especially those with lung cancer. Non-small-cell lung cancer (NSCLC) patients with *EGFR* mutations have a 50%–70% risk of developing BM (1). Although the survival of patients with BM has improved over time, central nervous system (CNS) events remain the major source of morbidity and mortality.

At present, the standard non-invasive local treatment for BM is radiotherapy (RT). The new generation of molecularly targeted drugs are liposoluble compounds with small molecular weight, and the endogenous receptor-mediated transcytosis employs vesicular trafficking to transport ligands across the endothelium of the blood–brain barrier. Considering that the use of new targeted agents has resulted in good outcomes with well-tolerated toxicities, the treatment strategy for patients with BM might change in the era of targeted treatment. A few clinical studies reported that single-agent *EGFR*- tyrosine kinase inhibitors (TKIs) showed promising results in TKI treatment-naive patients (2, 3). Compared with crizotinib, brigatinib showed consistent superiority in intracranial objective response rate (iORR) (67%–78%) and progression-free survival (PFS) in patients with *ALK* rearrangement (4, 5). Furthermore, a few multi-target anti-angiogenesis agents have shown good response rates in clinical studies (6, 7). However, it remains unclear whether it is reasonable enough to defer RT until the intracranial progression is noted in patients on TKIs based on the aforementioned results, considering potential RT toxicity. In the era of targeted therapy, the optimal timing of intracranial RT and TKI treatment in patients with BM remains to be further confirmed.

Because no published phase III clinical trials have addressed this issue, we conducted a retrospective analysis to explore the optimal sequence of local RT and systemic TKI use in the treatment of BM and assessed the feasibility and toxicity of both treatments. Furthermore, we tested the hypothesis that administering RT as upfront treatment, followed by target therapy, is not inferior to reserving RT as salvage treatment for newly diagnosed BM.

## 2 Methods and Materials

### 2.1 Eligibility Criteria

We conducted a cohort study and retrospectively reviewed patients from October 2010 to October 2020. The eligibility criteria were as follows: (1) age ≥ 18 years; (2) Karnofsky performance score (KPS) ≥ 60, or KPS ≥ 40 but only caused by BM; (3) histologically proven primary NSCLC and newly diagnosed BM on contrast-enhanced MRI, which was measurable; (4) presence of gene-mutation targets and receipt of TKI treatment for more than 1 month; (5) and receipt of hypofractionated stereotactic RT (HFSRT) with or without whole-brain therapy (WBRT). The exclusion criteria included (1) synchronous or metachronous malignancies that might affect survival; (2) receipt of craniotomy as the initial treatment; (3) receipt of RT without dose prescription in detail; (4) presence of leptomeningeal metastases; (5) unfinished RT course; (6) receipt of RT alone, or palliative WBRT alone; or (7) incomplete sociodemographic and/or clinicopathologic baseline data.

The protocol of this trial was approved by the institutional ethics review board (approval number: 2021010711263002); given its retrospective nature, the need for informed consent was waived by the review board.

### 2.2 Treatments and Evaluation

Pretreatment evaluation was performed within 1 week before treatment and included a full medical history, physical and neurologic examinations, and laboratory investigations; brain MRI was routinely performed for all patients in both groups. All patients underwent weekly physical and neurologic examinations, as well as a complete blood count and blood biochemical examinations during concurrent or sequential treatment. The following variables were reviewed for analyses: age, sex, Karnofsky Performance Score (KPS) at the time of BM, date of initial cancer and BM diagnosis, histology of primary disease, gene mutation type, presence of BM symptoms, and extracranial disease status. BM number, BM volume, RT treatment regimens, name of TKI and date the first treatment for BM were documented in detail. A complete clinical evaluation, laboratory tests, and MRI were performed 1 to 2 months after treatment. The follow-up evaluations consisted of clinical evaluations, enhanced brain MRI, and imaging examinations of the primary tumor and extracranial metastases every 3 months. The first progression site, date, and salvage treatments after any progression were recorded. The most recent follow-up time and death were documented.

The iORR was evaluated based on the Response Assessment in Neuro-Oncology of Brain Metastases (RANO-BM) criteria (8). Toxicity was recorded during treatment with TKIs and RT, and late neurological treatment-emergent adverse event (AEs) were recorded before the intracranial progression events, according to the NRG-RTOG Acute and Late CNS Toxicity Criteria and the National Cancer Institute Common Terminology Criteria for Adverse Events (version 4.0).

### 2.3 Endpoints

The endpoints included overall survival (OS), brain metastasis-specific survival (BMSS), intracranial progression-free survival (iPFS), iORR, and toxicities. OS was derived from the date of BM diagnosis until death from any cause or censored on the last follow-up. BMSS was defined as the interval from BM diagnosis to death resulting from BM or censored on the last follow-up. iPFS was defined as the interval from BM diagnosis to intracranial progression (including the growth of a previous lesion or the development of a new lesion), or death due to intracranial progression. iORR was defined as the percentage of patients who showed intracranial complete and partial response.

### 2.4 Statistical Analysis

The patients’ characteristics and iORR in different groups were compared using chi-square test or Fisher’s exact test for categorical variables, and Wilcoxon or Kruskal–Wallis H rank-sum test of variance for continuous data.

OS, BMSS, and iPFS were calculated using Kaplan–Meier plots. The log-rank test was used to assess for differences. Cox proportional hazards regression was performed for multivariable analysis in the entire patient group and all subgroups. Univariate and multivariate Fine-Grey competing risk regression was performed with the endpoint of intracranial progression, in the context of the competing risk of death (9). Significance for inclusion in the multivariate model was set at *p* < 0.10 and *p* < 0.05 was considered as a significant predictor of outcomes.

All analyses were performed using the SPSS (version 26.0, IBM Corporation, Armonk, NY) and R software package (version 4.0.3, http://www.r-project.org/).

## 3 Results

### 3.1 Patient Characteristics

**Figure 1:**
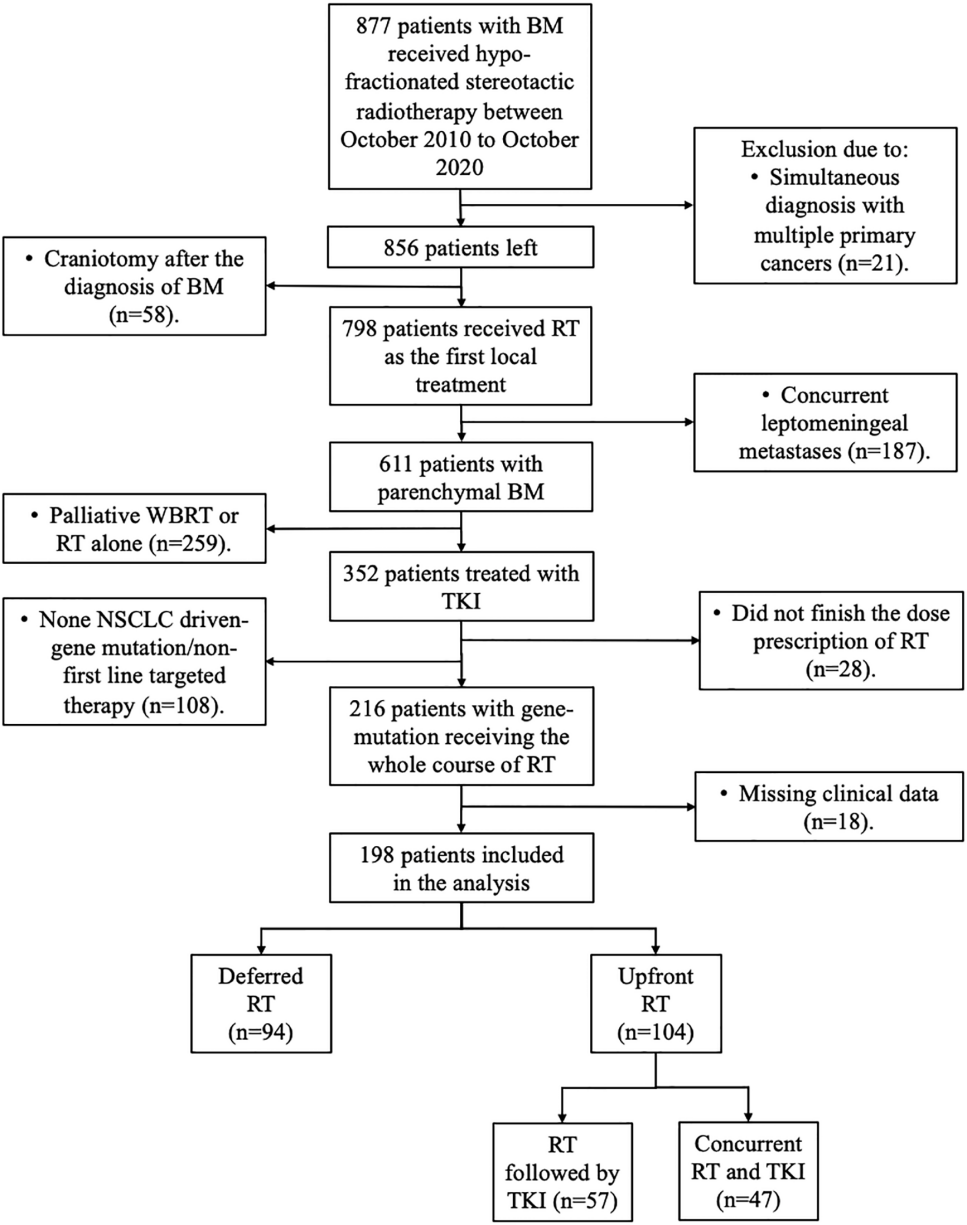
Flow diagram showing patient selection.

Between October 2010 and October 2020, 899 patients were newly diagnosed and referred to receive HFSRT with or without WBRT at our institution. A total of 198 patients were finally included in the study (**Figure 1**): 94 (47.5%) patients first received target therapy followed by RT (deferred RT group), and 104 patients first received RT followed by target therapy (57, 28.8%) or concurrent RT and target therapy (47, 23.7%) (upfront RT group).

Patient baseline characteristics are listed in **Table 1**. The median age of the entire cohort was 54 years (range, 29–83). The median BM number and volume were 3 (range, 1–39) and 3.7 cc (range, 0.01–103.9). The median time from primary disease to BM was 10.5 months (range, 0.0–115.9). The median time from BM to first RT was 8.3 months (range, 1.1–40.8) and 0.7 months (0.0–5.9) for the deferred and upfront RT groups. More patients were treated with upfront RT in the early 5 years. The main clinical characteristics between the deferred and upfront RT group were similar, except for BM at diagnosis, symptomatic BM and TKI before BM.

**Table 1:** Clinical characteristics of patients in the deferred RT and upfront RT groups.

Characteristic	Total	Deferred RT (*n* = 94)	Upfront RT (*n* = 104)	*p*-value
		*N* (%)	*N* (%)	
Year of treatment				<0.001
2010–2015	94 (47.5)	23 (24.5)	71 (68.3)	
2016–2020	104 (52.5)	71 (75.5)	33 (31.7)	
Age at BM (median, years)	54	53	54	0.792
Sex				0.709
Male	87 (43.9)	40 (42.6)	47 (45.2)	
Female	111 (56.1)	54 (57.4)	57 (54.8)	
KPS				0.145
<80	31 (15.7)	11 (11.7)	20 (19.2)	
≥80	167 (84.3)	83 (88.3)	84 (80.8)	
Ds-GPA				0.308
0–2	119 (60.1)	60 (63.8)	59 (56.7)	
2.5–4.0	79 (39.9)	34 (36.2)	45 (43.3)	
Symptomatic BM				<0.001
Yes	80 (40.4)	21 (22.3)	59 (56.7)	
No	118 (59.6)	73 (77.7)	45 (43.3)	
BM number				0.238
1	62 (31.3)	30 (31.9)	32 (30.8)	
2–4	71 (35.9)	26 (27.7)	45 (43.3)	
≥5	65 (32.8)	38 (40.4)	27 (26.0)	
BM volume				0.543
<5 cc	122 (61.6)	60 (63.8)	62 (59.6)	
≥5 cc	76 (38.4)	34 (36.2)	42 (40.4)	
Extracranial disease				0.268
Primary/regional site	43 (21.7)	17 (18.1)	26 (25.0)	
Metastases	26 (13.1)	20 (21.3)	6 (5.8)	
Both	90 (45.5)	43 (45.7)	47 (45.2)	
None	39 (19.7)	14 (14.9)	25 (24.0)	
Extracranial disease status				0.045
Progressed	68 (34.3)	38 (40.4)	30 (28.8)	
Controlled	91 (46.0)	42 (44.7)	49 (47.1)	
None	39 (19.7)	14 (14.9)	25 (24.0)	
BM at diagnosis				0.013
Synchronous	59 (29.8)	20 (21.3)	39 (37.5)	
Metachronous	139 (70.2)	74 (78.7)	65 (62.5)	
Time to BM				0.840
<36 m	176 (88.9)	84 (89.4)	92 (88.5)	
≥36 m	22 (11.1)	10 (10.6)	12 (11.5)	
TKI before BM				<0.001
Yes	58 (29.3)	39 (41.5)	19 (18.3)	
No	140 (70.7)	55 (58.5)	85 (81.7)	
Gene-mutation type				
*EGFR* mutation	159 (80.3)	72 (76.6)	87 (83.7)	0.212
Recorded mutation	123 (62.1)	69 (73.4)	54 (51.9)	
Uncertain	36 (18.2)	3 (3.2)	33 (31.7)	
*ALK* rearrangements	32 (16.2)	20 (21.3)	12 (11.5)	0.063
Other mutations	7 (3.5)	2 (2.1)	5 (4.8)	0.308
* RET* fusion	3 (1.5)	1 (1.1)	2 (1.9)	
* C-MET* mutations	2 (1.0)	0 (0.0)	2 (1.9)	
* ROS-*1 rearrangements	1 (0.5)	0 (0.0)	1 (1.0)	

N, number; BM, brain metastases; KPS, Karnofsky Performance Score; Ds-GPA, disease specific-graded prognostic assessment; TKI, tyrosine kinase inhibitor; RT, radiation therapy; EGFR, epidermal growth factor receptor; ALK, anaplastic lymphoma kinase; C-MET, cellular–mesenchymal epithelial transition factor; ROS-1, c-Ros Oncogene 1.

### 3.2 Treatments

RT was performed using the Brainlab planning system, Varian linear accelerator, and helical tomotherapy system. The techniques included stereotactic RT (*n* = 96), intensity-modulated RT (*n* = 2), volumetric modulated arc therapy (*n* = 35), and tomotherapy (*n* = 65). Treatment regimens for patients were chosen according to a risk-prognosis model (10). The regimens in the upfront RT group included upfront SRS (*n* = 113) and WBRT (*n* = 85). WBRT was first used in 49 (47.1%) patients in the upfront RT group and 36 (38.3%) patients in the deferred RT group (*p* = 0.211). The median fraction schedule was determined as follows: 50–52.5 Gy/10–15 fraction (f) for large (≥5 cc) lesions, 32–42 Gy/4–7 f for lesions close to the functional area, 40–45 Gy/10–15 f for lesions within the brainstem, 20–36 Gy/1–3 f for single small focus, 60 Gy/15–20 f for other concurrent small lesions, 40 Gy/20 f for WBRT concurrent with HFSRT, and 30 Gy/10 f for WBRT followed by HFSRT. Fifty-eight patients received a simultaneous integrated boost (SIB).

**Supplementary Table S1** summarizes the dose prescriptions and their corresponding biologically effective doses, equivalent doses in 2 Gy/f and the effects of response.

### 3.3 Outcomes

#### 3.3.1 iORR

The iORR in the entire cohort was 59.1%. In the deferred RT group, 38 (40.4%) patients showed complete and partial responses, 37 (39.4%) patients showed stable disease, and 19 (20.2%) showed progressed disease. The corresponding numbers were 79 (76.0%), 18 (17.3%), and 7 (6.7%) in the upfront RT group, respectively (*p* < 0.001).

#### 3.3.2 Patterns of Failure

Until the last follow-up, 182 (91.9%) of the 198 patients experienced intracranial and/or extracranial progression. A total of 71 (75.5%) and 47 patients (45.2%) developed intracranial cranial failure in the deferred RT and the upfront RT group, respectively. As shown in **Table 2**, the deferred RT group had a higher rate of intracranial failure alone than the upfront RT group (72.3% *vs.* 37.5%, *p* < 0.001), while the upfront RT group showed a higher rate of extracranial failure alone (47.1% *vs.* 16.0%, *p* < 0.001). Among patients who showed intracranial progression alone, the upfront RT group showed a higher rate of intracranial distant failure than the deferred RT group (46.2% *vs.* 26.5%, *p* = 0.020).

**Table 2:** Patterns of failure in the entire cohort.

Site of the first progression	Deferred RT (*n* = 94)	Upfront RT (*n* = 104)	*p*-value
	*N* (%)	*N* (%)	
Intracranial site alone	68 (72.3)	39 (37.5)	<0.001
Local	33 (48.5)	15 (38.5)	0.314
Distant	18 (26.5)	18 (46.2)	0.020
Concurrent	16 (23.5)	6 (15.4)	0.316
None	1 (1.5)	0 (0.0)	0.532
			
Extracranial site alone	15 (16.0)	49 (47.1)	<0.001
Primary	2 (13.3)	4 (8.2)	0.618
Regional	3 (20.0)	5 (10.2)	0.377
Metastases	6 (40.0)	26 (53.1)	0.376
Concurrent	4 (26.7)	14 (28.6)	1.000
			
Both intra and extra cranial site	3 (3.2)	8 (7.7)	0.167
			
None	8 (8.5)	8 (7.7)	0.637

N, number; BM, brain metastases; KPS, Karnofsky Performance Score; TKI, tyrosine kinase inhibitor; RT, radiation therapy.

#### 3.3.3 Survival Outcomes

**Figure 2:**
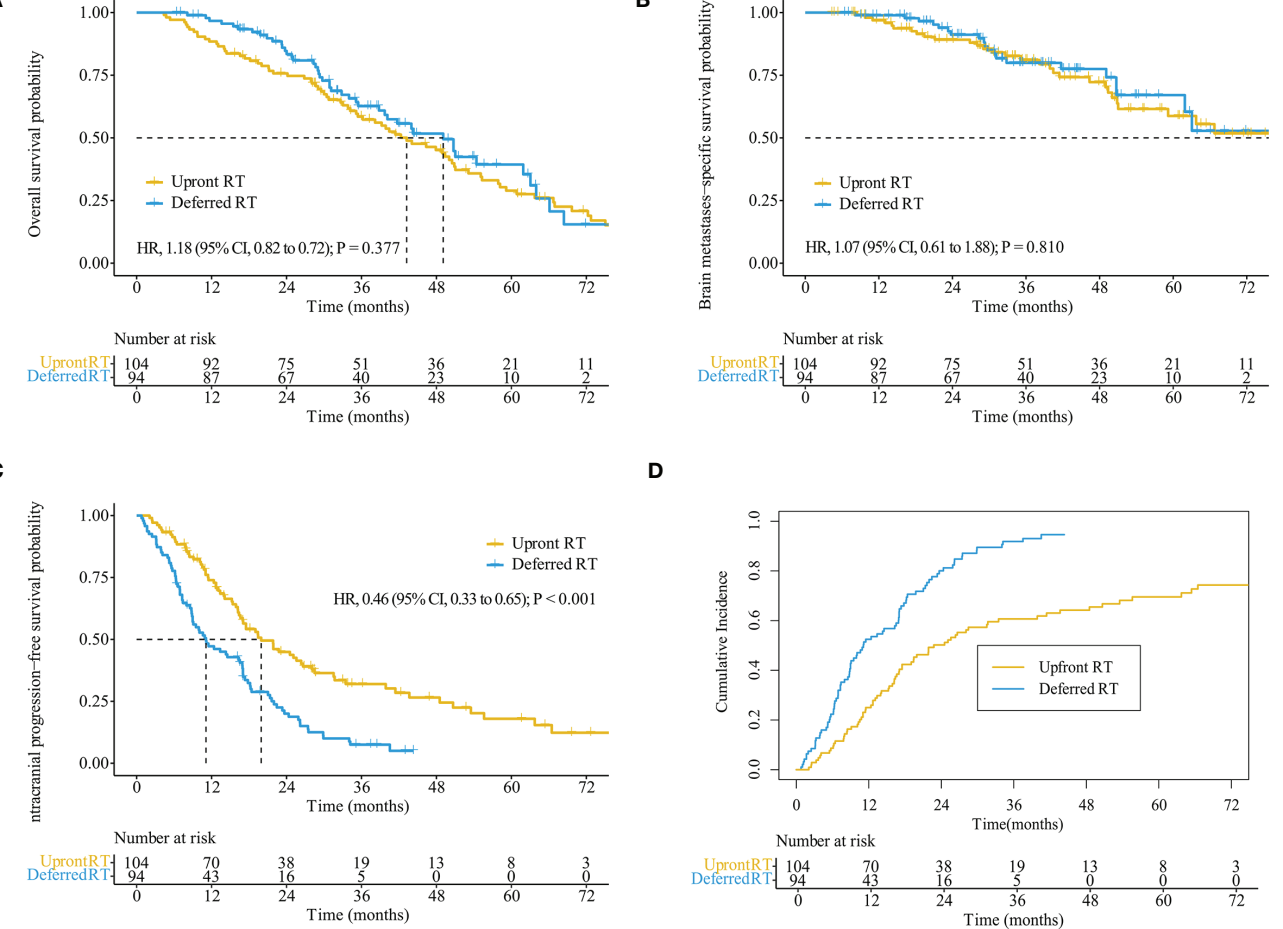
Kaplan–Meier analysis comparing survival stratified by treatment regimens in the entire cohort. Overall survival **(A)**, brain metastases-specific survival **(B)**, intracranial progression-free survival **(C)**, and cumulative incidence of intracranial progression using competing risks regression analysis **(D)**.

**Figure 3:**
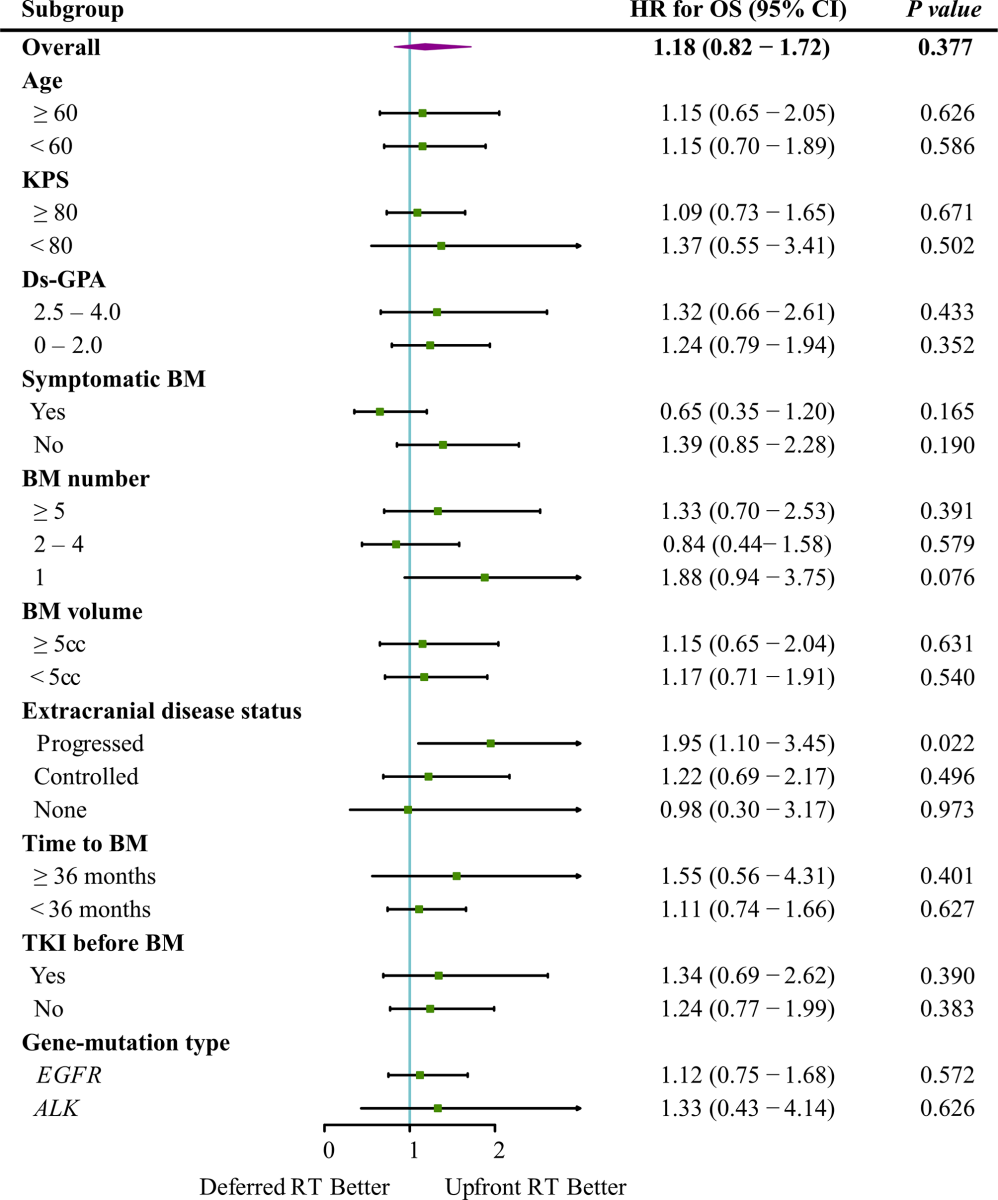
Forest plot depicting the HRs of deferred RT *vs.* upfront RT regimens in different subgroups.

**Figure 4:**
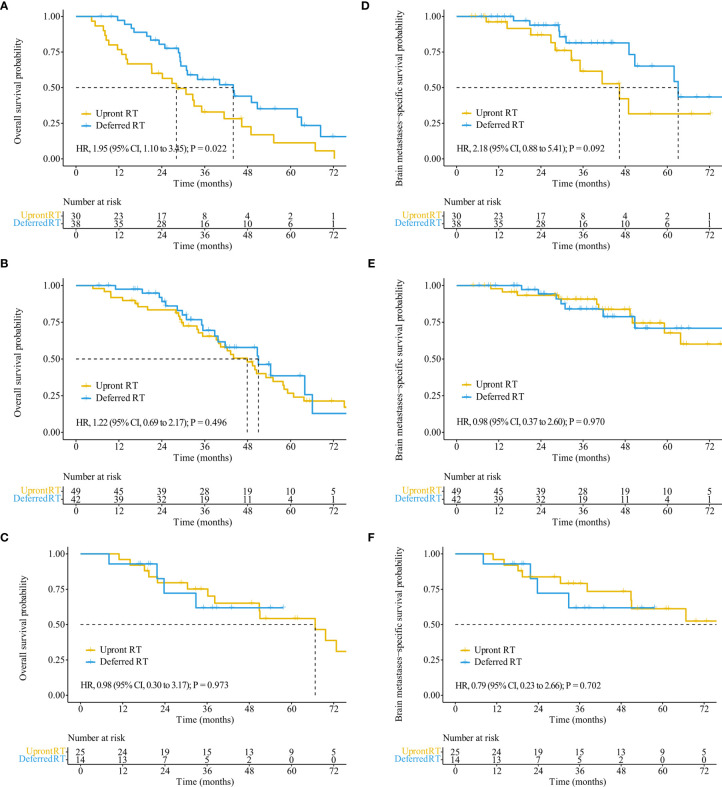
OS and BMSS stratified by treatment regimens in patients with different extracranial disease statuses. OS **(A)** and BMSS **(D)** of patients with progressed extracranial disease. OS **(B)** and BMSS **(E)** of patients with controlled extracranial disease. OS **(C)** and BMSS **(F)** of patients with non-extracranial disease.

The median follow-up time for all patients was 55.7 months (interquartile range, 21.8–50.8 months). Of the 122 patients who died, 65 (53.3%) died of extracranial metastases, 43 (35.2%) died of BM progression, 13 (10.7%) died of internal medical diseases, and one (0.8%) met with an accident. The median survival time (MST) for the entire cohort was 44.2 months [95% confidence interval (CI): 37.2–51.3 months]. OS did not differ significantly between the deferred RT and upfront RT groups, with a MST of 49.1 months (95% CI, 38.9–59.2 months; *p* = 0.377) months and 43.2 months (95% CI, 34.2–52.2 months; **Figure 2A**) months. After the Cox proportional hazards analysis, age (≥60 years; HR, 1.55; 95% CI, 1.06–2.28; *p* = 0.025), BM volume (≥5 cc; HR, 1.54; 95% CI, 1.01–2.33; *p* = 0.044), progressed extracranial disease (controlled: HR, 0.66; 95% CI, 0.44–0.99, *p* = 0.044; none: HR, 0.40; 95% CI, 0.23–0.72, *p* = 0.002), and gene mutation type (others: HR, 4.09; 95% CI, 1.45–11.55, *p* = 0.008) were factors influencing OS (**Table 3**). For patients with progressed extracranial disease (HR, 1.95; 95% CI, 1.10–3.45; *p* = 0.022; **Figure 3**), MST was longer in the deferred RT group (**Figure 4A**). No OS difference was observed between two groups for patients with controlled or non-extracranial disease (**Figures 4B, C**).

**Table 3:** Univariable and multivariable analyses of variables associated with OS in the entire group.

Variables	Univariable Analysis			Multivariable Analysis		
	HR	95% CI	*p*	HR	95% CI	*p*
Years of treatment						
2010–2015		Reference				
2016–2020	0.93	0.62–1.39	0.710	–	–	–
Age at BM, years						
≥60 *vs.* <60	1.71	1.19–2.47	0.004	1.55	1.06–2.28	0.025
Sex						
Male *vs.* female	1.05	0.73–1.51	0.797	–	–	–
KPS						
≥80 *vs.* <80	0.67	0.43–1.06	0.087	1.06	0.63–1.78	0.824
GPA						
2.5–4.0 *vs.* 0–2	0.67	0.46–0.99	0.041	–	–	–
Symptomatic BM						
Yes *vs.* no	1.37	0.96–1.97	0.084	1.32	0.90–1.95	0.160
BM number						
≥5		Reference		–	–	–
2–4	0.79	0.50–1.26	0.322	–	–	–
1	0.73	0.47–1.13	0.159	–	–	–
BM volume						
≥5 cc *vs.* <5 cc	1.73	1.20–2.49	0.003	1.54	1.01–2.33	0.044
Extracranial disease status						
Progressed		Reference			Reference	
Controlled	0.59	0.40–0.88	0.009	0.66	0.44–0.99	0.044
None	0.44	0.25–0.75	0.003	0.40	0.23–0.72	0.002
Time to BM						
≥36 m *vs.* <36 m	1.54	0.90–2.64	0.113	–	–	–
TKI before BM						
Yes *vs.* no	1.30	0.88–1.92	0.194	–	–	–
Gene-mutation type						
*EGFR*		Reference			Reference	
*ALK*	0.57	0.32–1.02	0.058	0.61	0.33–1.11	0.103
Others	2.85	1.03–7.85	0.043	4.09	1.45–11.55	0.008
Treatment regimens						
Upfront TKI		Reference		–	–	–
Upfront SRS	1.24	0.80–1.91	0.340	–	–	–
Upfront WBRT	1.11	0.73–1.75	0.572	–	–	–

BM, brain metastases; KPS, Karnofsky Performance Score; TKI, tyrosine kinase inhibitor; RT, radiation therapy; WBRT, whole brain radiotherapy; SRS, stereotactic radiosurgery; HFSRT, hypofractionated stereotactic radiotherapy; CI, confidence interval; HR, hazard ratios.

The median BMSS time for the entire cohort was 82.9 months (95% CI, 58.9–106.9 months). BMSS did not differ significantly between the deferred RT and upfront RT groups, with a median time of 82.9 months (95% CI, 59.4–106.4 months; *p* = 0.810) and 92.1 months (95% CI, 48.5–135.6 months; **Figure 2B**). After controlling for competing risk factors (death due to non-BM causes) with the Fine-Gray competing risks regression model, the upfront RT group still showed a similar probability of BMSS as the deferred RT group, with adjusted sub-distribution hazard ratios (SHRs) of 1.06 (95% CI, 0.61–1.83; *p* = 0.850). After the Cox proportional hazards analysis, extracranial disease status (controlled: HR, 0.58; 95% CI, 0.31–1.10, *p* = 0.095) was the factor nearly influencing BMSS (**Supplementary Table S2**). For patients showing progressed extracranial disease, the median BMSS was nearly longer in the deferred RT group (HR, 2.18; 95% CI, 0.88–5.41; *p* = 0.092; **Figure 4D**). No BMSS difference was observed between two groups for patients with controlled or non-extracranial disease (**Figures 4E, F**).

#### 3.3.4 Intracranial Progression

The median iPFS for the entire cohort was 16.9 months (95% CI, 14.9–18.9 months). The median iPFS for the deferred RT and upfront RT groups was 11.1 months (95% CI, 6.7–15.5 months) and 19.9 months (95% CI, 14.9–25.0 months), respectively (*p* < 0.001, **Figure 2C**). After controlling for competing risk factors (death before any intracranial progress) with the Fine-Gray competing risks regression model, the upfront RT group still showed a significantly lower probability of intracranial progression, with an adjusted SHR of 0.41 (95% CI: 0.30–0.57; *p* < 0.001, **Figure 2D**). After the Cox proportional hazards analysis, upfront RT (upfront SRS: HR, 0.49; 95% CI, 0.32–0.74; upfront WBRT: HR, 0.40, 95% CI, 0.27–0.59; *p* < 0.001) and extracranial disease status (controlled: HR, 0.64; 95% CI, 0.50–1.03, *p* = 0.072; none: HR, 0.54; 95% CI, 0.33–0.90, *p* = 0.019) were the factors significantly related to longer iPFS (**Table 4**).

**Table 4:** Univariable and multivariable analyses of prognostic variables for iPFS in the entire group (competing risks regression analysis).

Variables	Univariable Analysis			Multivariable Analysis		
	HR	95% CI	*p*	HR	95% CI	*p*
Years of treatment						
2010–2015		Reference				
2016–2020	0.70	0.51–0.96	0.026	0.97	0.69–1.36	0.850
Age at BM, years						
≥60 *vs.* <60	1.18	0.85–1.67	0.350	–	–	–
Sex						
Male *vs.* female	0.85	0.62–1.18	0.340	–	–	–
KPS						
≥80 *vs.* <80	1.14	0.74–1.77	0.540	–	–	–
GPA						
2.5–4.0 *vs.* 0–2	1.19	0.86–1.64	0.290			
Symptomatic BM						
Yes *vs.* no	1.24	0.89–1.73	0.210	–	–	–
BM number						
≥5		Reference		–	–	–
2–4	1.02	0.70–1.49	0.910	–	–	–
1	0.93	0.62–1.38	0.720	–	–	–
BM volume						
≥5 cc *vs.* <5 cc	1.20	0.87–1.66	0.260	–	–	–
Extracranial disease status						
Progressed		Reference			Reference	
Controlled	0.70	0.49–1.00	0.049	0.72	0.50–1.03	0.072
None	0.51	0.31–0.83	0.006	0.54	0.33–0.90	0.019
Time to BM						
≥36 m *vs.* <36 m	1.50	0.82–2.73	0.190	–	–	–
TKI before BM						
Yes *vs.* no	0.83	0.59–1.18	0.310	–	–	–
Gene-mutation type						
*EGFR*		Reference			Reference	
*ALK*	1.38	0.95–2.01	0.093	1.25	0.84–1.85	0.270
Others	0.80	0.26–2.47	0.700	1.01	0.31–3.32	0.990
Treatment regimens						
Upfront TKI		Reference			Reference	
Upfront SRS	0.43	0.29–0.65	< 0.001	0.49	0.32–0.74	<0.001
Upfront WBRT	0.40	0.27–0.58	< 0.001	0.40	0.27–0.59	<0.001

BM, brain metastases; KPS, Karnofsky Performance Score; TKI, tyrosine kinase inhibitor; RT, radiation therapy; WBRT, whole brain radiotherapy; SRS, stereotactic radiosurgery; HFSRT, hypofractionated stereotactic radiotherapy; CI, confidence interval; SHR, sub-distribution hazard ratios.

### 3.4 AEs

AEs possibly associated with the treatment are reported in **Supplementary Table S3**. Overall, TKI combined with RT was well tolerated. The rates of possible TKI-related systemic AEs were similar between groups. There were no statistically significant differences in terms of acute or late CNS toxicities. Forty-four patients (22.2%) reported acute neurological AEs, which appeared as various degrees of intracranial hypertension during treatment, most of which belonged to symptomatic BM. Only one patient had G4 acute neurological AEs, but showed resolution after completing the RT course. Forty-eight patients (24.2%) developed late CNS toxicity. Patients with G3 late CNS toxicity tended to have large tumor volume and symptoms before RT. After treatment, the symptoms were mostly relieved but reappeared several years later because of radiation necrosis or possible intracranial progression.

### 3.5 Salvage Treatments

Until the last follow-up, 150 out of 228 (65.8%) patients received salvage RT owing to intracranial local recurrent or distant failure, including 80 (80.8%) in the deferred RT group and 70 (54.3%) in the upfront RT group (*p* < 0.001). Of the 80 patients in the deferred RT group, 22 (27.5%) received re-irradiation. In all, 155 (78.3%) patients received salvage systemic treatment, including 84 (89.4%) in the deferred RT group and 71 (68.3%) in the upfront RT group (*p* < 0.001). Of the 84 patients in the deferred RT group, 58 (69.0%) received second-line TKI. The median time from BM diagnosis to salvage intracranial treatment did not differ significantly between groups (deferred RT: 30.3 months, 95% CI, 22.5–38.0 months; upfront RT: 21.9 months, 95% CI, 14.7–29.0 months; *p* = 0.183). Only 1 (1.1%) patient in the deferred RT group and 3 (2.9%) in upfront RT group received salvage craniotomy.

## 4 Discussion

In a direct comparison of upfront and deferred RT, we found that upfront RT may increase iPFS but did not significantly prolong the median OS or BMSS time. The deferred RT group showed survival advantages over the progressed extracranial disease subgroup. Both groups showed well-tolerated toxicities.

A few other studies (**Supplementary Table S4**) focused on the treatment sequence of EGFR-mutated NSCLC BM patients. Magnuson (11) reported that upfront RT improved survival outcomes more than upfront TKI (iPFS: 37.9 *vs.* 10.6 months, *p* < 0.001; MST: 34.1 *vs.* 19.4 months, *p* = 0.01), and upfront SRS showed the best response in the subgroup of ds-GPA 2.0–4.0. The follow-up study (12) also confirmed that upfront SRS showed a better MST than upfront WBRT or upfront TKI despite the prognosis status. Miyawaki (13) showed that only patients with one to four BMs had longer MST and iPFS time. One study compared the outcomes of RT with crizotinib or crizotinib alone and showed that early SRS had survival benefit for oligo-BM patients at baseline, but it could be deferred for symptomatic patients with multiple BM (14). Gerber (15) has also demonstrated that patients with a more favorable ds-GPA who received upfront SRS had a longer MST than those who received upfront WBRT and TKI. However, factors such as extracranial diseases status may have introduced biases between groups. Ds-GPA included extracranial metastases, but the previous studies did not estimate the primary and regional diseases status and determine whether the extracranial diseases was under control. Notably, some of the previous studies included patients who received craniotomy as local treatment (LT), and only approximately 50% of the patients in the upfront TKI group received subsequent LT or RT (12, 13). These factors may have influenced the survival results and underestimated the benefit of deferred RT.

Previous studies seemed to indicate that upfront SRS may provide greater iPFS and even OS benefits than upfront TKI, especially for patients with limited BM or those showing favorable results in the prognostic assessment. In this study, we focused on BM patients who received HFSRT with or without WBRT, and all patients in the deferred RT group received subsequent or salvage RT. The results showed a slightly less IPFS but a higher OS in the entire cohort than in previous studies (16, 17), and the upfront RT regimen reduced the intracranial cumulative incidence risk by more than 50% in comparison with deferred RT, but was not related to longer survival. This might be explained by the following reasons. First, the intracranial RT technique has changed substantially over the past decade. Proper RT strategies have been suggested to effectively prolong survival, especially for cases with complex BM (18). Since VMAT and tomotherapy with SIB have been routinely used after 2016, the majority (77.7% *vs.* 22.1%, *p* < 0.001) of patients recruited from 2010 to 2015 received SRS and two/three-dimensional WBRT without SIB. Second, osimertinib demonstrated greater penetration of the blood–brain barrier than gefitinib or afatinib (19), and showed benefits in terms of PFS in BM patients in comparison with the standard *EGFR*-TKIs (20). In addition, lorlatinib had also been confirmed to be much more effective for iORR and iPFS than crizotinib (21). However, most of the patients before 2016 received first-generation TKIs as initial targeted agents instead of third-generation TKIs (97.8% *vs.* 64.7%, *p* < 0.001). We had excluded the patients who received TKI alone and those with intracranial disease in a controlled status who did not receive RT, which would have reduced the IPFS in comparison with previous studies. Moreover, we found that progression of extracranial diseases was the most common cause of death and a solid factor for survival. About 60% of the patients in the entire patient population had extracranial diseases, and approximately 50% of the patients in the upfront RT group experienced extracranial failure. The higher extracranial tumor burden in this study may have influenced the survival outcomes and may explain why the improved IPFS in the upfront RT group did not translate into an OS benefit. This study also upheld the view that among the patients showing progression of extracranial disease, the deferred RT group showed better OS than the upfront RT group. The salvage RT for intracranial progression after initial TKI prolonged the period of adequate control, and helped patients achieve similar or even better MST than the RT group. However, few other therapies were effective after initial RT. A recent study (22) showed that the median duration of TKI > 14 months was an independent factor related to better OS (HR 0.17, 95% CI 0.10–0.30; *p* < 0.001), not only for patients with oligometastatic/oligoprogressive disease, but also for those with polymetastatic/polyprogressive disease. A randomized trial of osimertinib with or without SRS for *EGFR*-mutated NSCLC with BMs from the Trans-Tasman Radiation Oncology Group to compare the iPFS at 12 months is ongoing (NCT03497767).

We had previously explored the effectiveness of upfront SRS and deferred WBRT in BM patients with focus larger than 3 cm (23). The results showed that the use of SRS as the initial treatment while reserving WBRT as the salvage therapy in case of distant intracranial recurrence made about 50% of the patients avoid WBRT throughout their lives. Moreover, we established a risk-prognosis model of distant brain failure to choose the optimal RT regimen for BM patients (10). Several other studies have also suggested that WBRT cannot confer a survival benefit despite being effective in controlling intracranial progression (24, 25). Jiang (26) showed that WBRT combined with TKIs offered no survival benefit over TKIs alone in *EGFR*-mutation BM patients. In the present study, we considered that target therapy may decrease the risk of brain failure and could further postpone the use of WBRT as salvage treatment. The results suggested that upfront WBRT provided a similar survival prognosis as deferred WBRT in the targeted treatment era. Therefore, the oncologists should still give a careful consideration to WBRT as the initial treatment for gene-driven BM patients to avoid deterioration in the patients’ cognitive functions and health-related quality of life (27, 28).

Patients who received TKI before BM were included because, in most cases, the intracranial site was the only failure site and extracranial disease was well-controlled. Furthermore, the targeted agents were changed to the next-generation TKI under most circumstances. Therefore, we did not exclude these patients from the study and included the whole group in the final analysis, which might reflect the real-world clinical practice data more accurately and appropriately. The results of univariable analysis showed that it did not influence the survival as well.

Although our study showed promising results with different treatment regimens for BM patients, some limitations should be addressed. First, this is a retrospective study that has inherent biases despite our effort to narrow down the inclusion criteria. Second, we have to acknowledge that several patients used TKI as salvage treatment other than sequential treatment in the upfront RT group, considering it was quite difficult to distinguish the treatment purpose because of the possible simultaneous extracranial disease progression. Overall, the appropriate sequence of RT and new-generation TKI needs more clinical evidence.

## 5 Conclusion

The present study suggests that upfront RT is more effective than deferred RT for intracranial control in BM patients, but offers no significant survival benefit. Upfront TKI is recommended in patients with progressed-extracranial disease if close surveillance and timely salvage local therapy can be achieved. Upfront RT might also be recommended in patients with a heavy intracranial tumor burden that mainly influences the survival.

## Data Availability Statement

The original contributions presented in the study are included in the article/**Supplementary Material**. Further inquiries can be directed to the corresponding authors.

## Ethics Statement

The studies involving human participants were reviewed and approved by 2021010711263002. Written informed consent for participation was not required for this study in accordance with the national legislation and the institutional requirements.

## Author Contributions

Conception/design: JX, SW, and YL. Provision of study material or patients: SY, NB, JX, JL, and JY. Collection and/or assembly of data: SY, QL, YZ, NB, YM, KW, XH, XC, LD, WW, and RZ. Data analysis and interpretation: SY, NB, JX, and SW. Manuscript writing: SY. Final approval of manuscript: JX, SW, and YL. All authors contributed to the article and approved the submitted version.

## Funding

This work was supported by the National Key Projects of Research and Development of China (grant number 2016YFC0904600), the Beijing Hope Marathon Special Fund (grant number LC2011A07), and the Chinese Anticancer Association Professional Committee of Neuro-Oncology (grant number CSNO-2015-MSD01).

## Conflict of Interest

The authors declare that the research was conducted in the absence of any commercial or financial relationships that could be construed as a potential conflict of interest.

## Publisher’s Note

All claims expressed in this article are solely those of the authors and do not necessarily represent those of their affiliated organizations, or those of the publisher, the editors and the reviewers. Any product that may be evaluated in this article, or claim that may be made by its manufacturer, is not guaranteed or endorsed by the publisher.
