# Supplementary material for: The Sequence of Intracranial Radiotherapy and Systemic Treatment With Tyrosine Kinase Inhibitors for Gene-Driven Non-Small Cell Lung Cancer Brain Metastases in the Targeted Treatment Era: A 10-Year Single-Center Experience
Source: Front Oncol. 2021 Oct 14;11:732883. doi: 10.3389/fonc.2021.732883 (PMC8552495; doi:10.3389/fonc.2021.732883)
Supplement: Supplementary file 1 [file Table_1.docx]

Supplemental Table 1. Hypofractionation schedules and relative biological equivalent doses

| Prescriptions | Deferred RT  N (%) | Upfront RT  N (%) | *P value* | BED (Gy) | EQD2 (Gy) | CR | PR | SD | PD |
| --- | --- | --- | --- | --- | --- | --- | --- | --- | --- |
| 20 Gy/1 f | 7 (7.4) | 11 (10.6) |  | 60.0 | 50.0 | 0 | 9 | 6 | 3 |
| 24 Gy/1 f | 7 (7.4) | 8 (7.7) |  | 81.6 | 68.0 | 2 | 5 | 6 | 2 |
| 24 Gy/2 f | 3 (3.2) | 4 (3.8) |  | 52.8 | 44.0 | 2 | 5 | 0 | 0 |
| 32 Gy/4 f | 0 (0.0) | 3 (2.9) |  | 57.6 | 48.0 | 0 | 3 | 0 | 0 |
| 36 Gy/3 f | 4 (4.3) | 6 (5.8) |  | 79.2 | 66.0 | 1 | 3 | 4 | 2 |
| 40 Gy/8 f | 6 (6.4) | 5 (4.8) |  | 60.0 | 50.0 | 2 | 6 | 3 | 0 |
| 40 Gy/10 f | 2 (2.1) | 3 (2.9) |  | 56.0 | 46.7 | 0 | 4 | 0 | 1 |
| 42 Gy/7 f | 0 (0.0) | 3 (2.9) |  | 67.2 | 56.0 | 0 | 2 | 0 | 1 |
| 45 Gy/15 f | 2 (2.1) | 3 (2.9) |  | 58.5 | 48.8 | 0 | 2 | 2 | 1 |
| 50 Gy/10 f | 5 (5.3) | 2 (1.9) |  | 75.0 | 62.5 | 0 | 0 | 5 | 2 |
| 52 Gy/13 f | 15 (16.0) | 18 (17.3) |  | 72.8 | 60.7 | 2 | 16 | 12 | 3 |
| 52.5 Gy/15 f | 7 (7.4) | 17 (16.3) |  | 70.9 | 59.1 | 1 | 18 | 4 | 1 |
| 60 Gy/20 f | 36 (38.3) | 21 (20.2) |  | 78.0 | 65.0 | 5 | 29 | 13 | 10 |
| Total | 94 | 104 | 0.112 | - | - | 15 | 102 | 55 | 26 |

Abbreviations: N, number; f, fraction; RT, radiation therapy; BED, biological equivalent dose (α/β = 10); EQD2, equivalent dose converted to 2 Gy per fraction (α/β = 10); CR, complete response; PR, partial response; SD, stable disease; PD, progressive disease

Supplemental Table 2 Univariable and multivariable analyses of variables associated with BMSS in the entire group (competing risk analysis)

| Variables | Univariable Analysis | | | Multivariable Analysis | | |
| --- | --- | --- | --- | --- | --- | --- |
|  | SHR | 95% CI | *P* | SHR | 95% CI | *P* |
| Years of treatment |  |  |  |  |  |  |
| 2010 – 2015 |  | Reference |  |  |  |  |
| 2016 – 2020 | 0.75 | 0.43-1.31 | 0.320 | - | - | - |
| Age at BM, years |  |  |  |  |  |  |
| ≥ 60 vs.＜60 | 0.87 | 0.50-1.53 | 0.630 | - | - | - |
| Sex |  |  |  |  |  |  |
| Male vs. female | 1.28 | 0.72-2.28 | 0.400 | - | - | - |
| KPS |  |  |  |  |  |  |
| ≥ 80 vs.＜80 | 0.96 | 0.45-2.03 | 0.910 | - | - | - |
| Ds-GPA |  |  |  |  |  |  |
| 2.5 – 4.0 vs. 0 – 2 | 0.75 | 0.43-1.29 | 0.290 | - | - | - |
| Symptomatic BM |  |  |  |  |  |  |
| Yes vs. no | 0.95 | 0.55-1.65 | 0.850 | - | - | - |
| BM number |  |  |  |  |  |  |
| ≥ 5 |  | Reference |  | - | - | - |
| 2 – 4 | 0.92 | 0.47-1.79 | 0.800 | - | - | - |
| 1 | 1.09 | 0.55-2.15 | 0.810 | - | - | - |
| BM volume |  |  |  |  |  |  |
| ≥ 5cc vs. < 5cc | 0.71 | 0.41-1.23 | 0.220 | - | - | - |
| Extracranial disease status |  |  |  |  |  |  |
| Progressed |  | Reference |  |  |  |  |
| Controlled | 0.58 | 0.31-1.10 | 0.095 | - | - | - |
| None | 1.34 | 0.69-2.61 | 0.390 | - | - | - |
| Time to BM |  |  |  |  |  |  |
| ≥ 36m vs.＜36m | 0.72 | 0.33-1.61 | 0.420 | - | - | - |
| TKI before BM |  |  |  |  |  |  |
| Yes vs. no | 1.14 | 0.61-2.14 | 0.680 | - | - | - |
| Gene mutation type |  |  |  |  |  |  |
| *EGFR* |  | Reference |  | - | - | - |
| *ALK* | 0.88 | 0.39-1.99 | 0.760 | - | - | - |
| Others | - | - | - | - | - | - |
| Treatment regimens |  |  |  |  |  |  |
| Upfront TKI |  | Reference |  | - | - | - |
| Upfront SRS | 1.21 | 0.64-2.27 | 0.560 | - | - | - |
| Upfront WBRT | 0.91 | 0.46-1.80 | 0.780 | - | - | - |

Abbreviations: BM, brain metastases; KPS, Karnofsky Performance Score; GPA, graded prognostic assessment; TKI, tyrosine kinase inhibitor; RT, radiation therapy; WBRT, whole brain radiotherapy; SRS, stereotactic radiosurgery; HFSRT, hypofractionated stereotactic radiotherapy; NSCLC, non-small cell lung cancer; BC, breast cancer; CI, confidence interval; SHR, sub-distribution hazard ratios.

Supplemental Table 3 Toxicities and evaluation grades among different groups

| Events | Grade | Deferred RT  N (%) | | Upfront RT  N (%) | | *P value* |
| --- | --- | --- | --- | --- | --- | --- |
| Leukopenia | Grade1-2 | 15 | (15.9) | 12 | (11.5) | 0.311 |
|  | Grade3-4 | 0 | (0) | 0 | (0) |  |
| Neutropenia | Grade1-2 | 7 | (7.5) | 5 | (4.8) | 0.432 |
|  | Grade3-4 | 1 | (1.1) | 1 | (1.0) |  |
| Anemia | Grade1-2 | 6 | (6.4) | 9 | (8.7) | 0.537 |
|  | Grade3-4 | 0 | (0) | 0 | (0) |  |
| Thrombocytopenia | Grade1-2 | 3 | (3.2) | 7 | (6.7) | 0.485 |
|  | Grade3-4 | 1 | (1.1) | 0 | (0) |  |
| Hypohepatia | Grade1-2 | 12 | (12.7) | 8 | (7.7) | 0.155 |
|  | Grade3-4 | 1 | (1.1) | 0 | (0) |  |
| Renal failure | Grade1-2 | 9 | (9.6) | 7 | (6.7) | 0.465 |
|  | Grade3-4 | 0 | (0) | 0 | (0) |  |
| Nausea/  vomiting | Grade1-2 | 21 | (22.4) | 30 | (28.8) | 0.251 |
|  | Grade3-4 | 0 | (0) | 0 | (0) |  |
| Acute neurologic AE | Grade1-2 | 24 | (26.1) | 16 | (15.4) | 0.175 |
|  | Grade3-4 | 1 | (1.1) | 3 | (2.9) |  |
| Late neurologic AE | Grade1-2 | 7 | (7.4) | 12 | (11.5) | 0.199 |
|  | Grade3-4 | 12 | (12.8) | 17 | (16.3) |  |

Abbreviations: N, number; TKI, tyrosine kinase inhibitor; RT, radiation therapy; AE, adverse event.Supplemental Table 4 Comparison of previous studies and the present study in sequence between radiation therapy and targeted therapy

| Study | N | Treatment | Follow up (m) | ORR | iPFS (m) | MST (m) | ≥ Grade3 toxicities |
| --- | --- | --- | --- | --- | --- | --- | --- |
| Magnuson, 2017 | 351  EGFR mutated NSCLC | Upfront SRS vs. Upfront WBRT vs. Upfront TKI | 22 (IQR 13 – 35) | NA | 23 vs. 24 vs. 17  P = 0.025 | 46 vs. 30 vs. 25  P < 0.001 | NA |
| Miyawaki, 2019 | 176  EGFR mutated NSCLC | Upfront RT/surgery±TKI vs. Upfront TKI | 23 (2.3 – 91) | NA | 22 vs. 12  P < 0.01  1 – 4 BM: 24 vs. 16  P < 0.01 | 28 vs. 23  P = 0.12  1 – 4 BM: 25 vs. 23  P = 0.02 | NA |
| Wang, 2019 | 93  EGFR mutated NSCLC | Upfront RT vs. TKI alone | 37.7 (3.4 – 63.2) | 94.3% vs. 85.2%  P = 0.093 | 27.6 vs. 16.1  P = 0.053 | 35.4 vs. 35.8  P = 0.695 | NA |
| Borghetti, 2019 | 106 EGFR/  ALK mutatedNSCLC (49 with brain RT) | RT before vs. after vs. concomitant to TKI | 9.1 (1.0 – 68.0) | NA | NA | 23  (1 year-OS: 62.7% vs. 88.9% vs. 79%, NS)  TKI > 14 m had better OS (HR 0.17, P < 0.001) | 4.1% |
| Chen, 2020 | 954  NSCLC  (447 with RT) | CT/TKI + deferred RT vs. CT/TKI + early RT | 54.8 (IQR 9.0 – 33.0) | NA | NA | Wild: 19.4 vs. 14.1  P < 0.001  EGFR/ALK+: 33.3 vs. 28.3 NS | NA |
| Our study | 198  gene-driven NSCLC BM | Upfront HFSRT±WBRT vs.  Deferred HFSRT±WBRT | 55.7 (IQR 21.8 – 50.8) | 76.0% vs.  40.4%  P < 0.001 | 19.9 vs.  11.1  P < 0.001 | 43.2 vs.  49.1  P = 0.377 | 20.2% vs.  17.2% |

Abbreviation: N, number; NSCLC, non-small cell lung cancer; BC, breast cancer; EGFR, epidermal growth factor receptor; RT, radiation therapy; TKI, tyrosine kinase inhibitor; WBRT, whole brain radiotherapy; SRS, stereotactic radiosurgery; HFSRT, hypofractionated stereotactic radiotherapy; CT, chemotherapy; IQR, Inter quartile range; ORR, objective response rate; iPFS, intracranial progression-free survival; MST, median overall survival time; OS, overall survival; NR, not available; NS, non-statistical significance
